# Integrative analysis of gene expression and copy number alterations using canonical correlation analysis

**DOI:** 10.1186/1471-2105-11-191

**Published:** 2010-04-15

**Authors:** Charlotte Soneson, Henrik Lilljebjörn, Thoas Fioretos, Magnus Fontes

**Affiliations:** 1 Centre for Mathematical Sciences, Lund University, Box 118, SE-221 00 Lund, Sweden; 2 Department of Clinical Genetics, Lund University Hospital, SE-221 85 Lund, Sweden

## Abstract

**Background:**

With the rapid development of new genetic measurement methods, several types of genetic alterations can be quantified in a high-throughput manner. While the initial focus has been on investigating each data set separately, there is an increasing interest in studying the correlation structure between two or more data sets. Multivariate methods based on Canonical Correlation Analysis (CCA) have been proposed for integrating paired genetic data sets. The high dimensionality of microarray data imposes computational difficulties, which have been addressed for instance by studying the covariance structure of the data, or by reducing the number of variables prior to applying the CCA. In this work, we propose a new method for analyzing high-dimensional paired genetic data sets, which mainly emphasizes the correlation structure and still permits efficient application to very large data sets. The method is implemented by translating a regularized CCA to its dual form, where the computational complexity depends mainly on the number of samples instead of the number of variables. The optimal regularization parameters are chosen by cross-validation. We apply the regularized dual CCA, as well as a classical CCA preceded by a dimension-reducing Principal Components Analysis (PCA), to a paired data set of gene expression changes and copy number alterations in leukemia.

**Results:**

Using the correlation-maximizing methods, regularized dual CCA and PCA+CCA, we show that without pre-selection of known disease-relevant genes, and without using information about clinical class membership, an exploratory analysis singles out two patient groups, corresponding to well-known leukemia subtypes. Furthermore, the variables showing the highest relevance to the extracted features agree with previous biological knowledge concerning copy number alterations and gene expression changes in these subtypes. Finally, the correlation-maximizing methods are shown to yield results which are more biologically interpretable than those resulting from a covariance-maximizing method, and provide different insight compared to when each variable set is studied separately using PCA.

**Conclusions:**

We conclude that regularized dual CCA as well as PCA+CCA are useful methods for exploratory analysis of paired genetic data sets, and can be efficiently implemented also when the number of variables is very large.

## Background

The abnormal behavior of cancer cells is in many cases caused by somatically acquired genetic alterations. Several types of genetic changes, such as fusion genes, mutations, copy number changes and abnormal methylation patterns, have been observed in malignant cells [1-4]. In most cases the alterations lead, either directly or indirectly, to changes in gene expression. The rapid development of oligonucleotide-based array platforms has enabled robust high resolution measurements of genetic alterations as well as gene expression. The initial focus has been on using the data from these methods separately, but there is an increasing interest in integrating different types of array-data generated from the same set of samples, e.g. by searching for correlated patterns in the two data sets. Mathematically, this aim can be formulated as finding the weighted linear combinations of variables from each of the two variable sets that show the highest correlation. The situation is complicated by the high dimensionality of microarray data sets, rendering the application of many classical statistical methods unfeasible.

The aim of this paper is to describe two methods for multivariate integrative analysis of paired data, based on Canonical Correlation Analysis (CCA [5]). Both methods put the main emphasis on the correlation structure of the data, and can be efficiently implemented also for data sets with a very large number of variables. First, we propose a new multivariate integrative method based on a regularized CCA, which is translated to its dual formulation to permit a computationally efficient implementation also when the number of variables is extremely large. Second, we describe the application of a classical CCA preceded by dimension reduction by Principal Components Analysis (PCA [6]). We evaluate the methods by applying them to a large paired data set consisting of copy number and gene expression measurements from 173 leukemia patients. Here we show that, without imposing prior knowledge, we are able to extract information which agrees well with previous knowledge of leukemia and extends beyond the results found when each variable set is analyzed separately with PCA. Furthermore we illustrate the advantage of emphasizing the correlation structure, as opposed to the covariance structure, of the data set.

CCA is a generalization of multivariate linear regression to the situation where there are more than one response variable. In its classical formulation CCA extracts a pair of features, each being a linear combination of the variables from one variable set, such that the correlation between the features is maximized. The classical formulation of CCA requires invertibility of the sample covariance matrices, making it impossible to apply e.g. to data sets where the number of variables exceeds the number of samples. Moreover it can be severely confounded by collinearities among the variables. To overcome this limitation Vinod [7] proposed a ridge regularized CCA where a multiple of the identity matrix was added to each of the covariance matrices. In regularized CCA, the criterion that is maximized by the extracted features is a penalized correlation, and more emphasis is put on extracting features explaining a large fraction of the variance in the respective variable sets. Full regularization, i.e. replacing the covariance matrices of the variable sets by identity matrices, discards the internal relations between the variables and yields Partial Least Squares regression (PLS [8]), which returns feature pairs with maximal covariance. PLS is computationally stable, even in cases where there are many or collinear variables, but the emphasis on covariance rather than correlation may lead to the extraction of feature pairs explaining a large fraction of the variance in each individual variable set, but only a small fraction of the correlation between them.

Several authors have addressed the integration of paired genetic data sets by posing specific questions, e.g. whether there are genes that are differentially expressed in samples possessing a certain copy number alteration, compared to samples without the alteration [9]. Thereby these authors adopt a "sequential" approach, in which one of the variable sets is used to stratify the study population, whereafter the other data set is analyzed in relation to this stratification [9-14]. Regression analyses, evaluating how the expression of each gene is affected by other types of genetic changes, have also been proposed [15,16], as well as studying all pairwise correlations between expression levels and copy numbers within a small set of known cancer-relevant genes [17]. Monni and Tadesse [18] considered a stochastic partitioning algorithm to identify subsets of co-regulated genes as well as subsets of predictor variables showing a similar influence on these genes. Different types of multivariate CCA- and PLS-based analysis methods have been proposed and applied for exploratory analysis and integration of genetic data sets. González *et al*. [19] applied the regularized CCA introduced by Vinod [7] to a paired nutrigenomic data set and a multidrug resistance data set. Moreover, several integrative CCA- and PLS-based methods imposing a sparse structure of the resulting feature vectors have been described [20-23]. CCA-based methods are symmetric in the two variable sets and the main objective is to find correlated features. This is in contrast to regression-based methods where the variables in one set are seen as predictors of those in the other set.

The regularized dual CCA described in this paper includes a ridge penalty on the covariance matrices. In this aspect it is similar to the method proposed by González *et al*. [19,24]. When the number of variables becomes very large solving the problem in the original formulation, as was done by González *et al*. [19,24], becomes computationally unfeasible. By translating the problem to its dual formulation where the computational complexity mainly depends on the number of samples, we achieve an efficient implementation also for very large data sets. Moreover, since the method proposed here is based on the dual formulation of CCA it can easily be transformed to search for nonlinear relationships by the kernel trick [25]. We keep the main emphasis on searching for correlated features also in the large data set context, which is one of the main differences compared to the sparse CCA-based methods [20-22], where full regularization of the CCA (i.e. replacement of the covariance matrices by identity matrices) is proposed to make computations feasible. The method proposed by Lê Cao *et al*. [23] is based on PLS and hence also covariance-maximizing. Focusing on correlation rather than covariance can be an advantage when the correlated features from the two variable sets do not contribute a large proportion of the variance. The features extracted by the regularized dual CCA will not be sparse, and penalties enforcing sparsity, such as LASSO constraints [26] or elastic net constraints [27], are not easily translated to the dual formulation. However, the features extracted by CCA are not in general used to interpret the biological relevance of the result since they are sensitive to collinearities [28]. Instead we interpret our results and receive a relevance ranking of the variables by the correlations between each variable and the extracted features.

Another approach towards constructing a multivariate integrative method keeping the emphasis on the correlation structure while being applicable to very large data sets is to use a classical CCA preceded by dimension reduction by PCA (discussed e.g. by Muller [29]). This is intuitively appealing since the PCA reduces the dimension in such a way that as much as possible of the variance is retained and returns uncorrelated features which can be imputed into the classical CCA. Furthermore, since both PCA and CCA can be expressed in a dual form, also the PCA+CCA can be efficiently implemented for large data sets, and by the kernel trick it can be generalized to search for various types of nonlinear relationships.

## Results

We apply regularized dual CCA and PCA+CCA to a paired data set of gene expression and SNP copy number measurements in 173 leukemia patients, representing ten different leukemia subtypes. The results are analyzed first by the relevance of the gene expression and copy number variables to the extracted features, and second by the representation of the samples in the space of the extracted features. Since the extracted features in this paper are used mainly for visualization, we consider only the first two pairs of features from each method. If the features are to be used for a more extensive interpretation, a careful choice on the dimension must be made, which in itself is a non-trivial matter.

We begin by splitting the data set into a tuning set and a validation set. The tuning set is used to estimate optimal regularization parameters and extract features, and the validation set is used only for visualization of the results and assessment of the generalization ability of the extracted features. The tuning set consists of two thirds of the samples, to provide a large enough basis for extraction of generalizable features, and the validation set consists of the remaining one third of the samples. The proportion of samples with a specific leukemia subtype is chosen to be similar in the two sets, otherwise the partition is random. The optimal regularization parameters for the regularized CCA are estimated using cross-validation on the tuning set. Using the optimal regularization parameters, we then extract the first two pairs of canonical features from the tuning set, and interpret their biological content using the cross-loadings of all variables with them. Finally, the extracted features are applied to the tuning set as well as the validation set, yielding two-dimensional representations of the samples. From these, we can extract groups of samples which are characterized by the extracted features, and assess the generalization ability of the features to the validation set. We compare the results from the optimally regularized dual CCA and PCA+CCA to those obtained by fully regularized dual CCA, un-regularized dual CCA, a sparse CCA method [21] and separate PCA of each variable set. For all methods, the features are extracted from the tuning set and applied to both the tuning and validation set. The low-dimensional representations of the validation set are shown in the paper, and the representations of the tuning set are given as Additional file 1: Supplementary Figures 1, 2, 3 and 4.

### Determination of optimal regularization parameters

**Figure 1:**
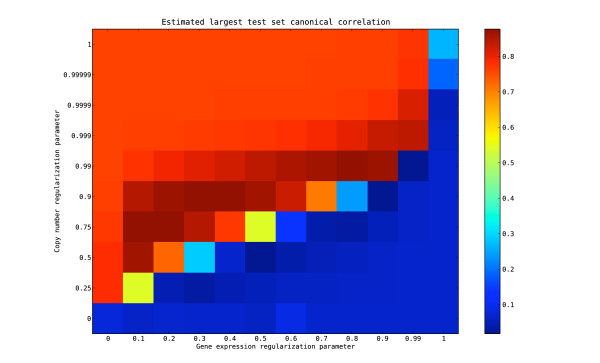
**Choosing the optimal regularization parameters**. Figure showing the estimated generalization ability of the first pair of extracted features from regularized dual CCA, for different combinations of regularization parameters *τ*_*x *_(regularization parameter for the copy number variables) and *τ*_*y *_(regularization parameter for the gene expression variables). The generalization ability is estimated as the correlation between the projection of a test set onto the first pair of features extracted from a training set, averaged over three cross-validation folds. The highest value,  = 0.877 is attained for *τ*_*x *_= 0.9 and *τ*_*y *_= 0.3.

The optimal regularization parameters for the two variable sets in the regularized dual CCA are considered to be those maximizing the generalization ability of the first extracted feature pair, which we define as the canonical correlation obtained when the extracted feature pair is applied to a test set. We use a 3-fold cross-validation strategy to estimate this canonical correlation (denoted ) from the tuning set, following partly the method introduced by Leurgans *et al*. [30], and subsequently applied by González *et al*. [19,24]. Figure 1 shows the value of  for different combinations of regularization parameters *τ*_*x *_(for the copy number data) and *τ*_*y *_(for the gene expression data). The largest value,  = 0.877, is attained for *τ*_*x *_= 0.9, *τ*_*y *_= 0.3. Apparently, the copy number data need more regularization than the gene expression data, which could be anticipated since many copy number variables are highly correlated.

Figure 1 also shows clearly that neither a model with no regularization (*τ*_*x *_= *τ*_*y *_= 0) nor a model with full regularization (*τ*_*x *_= *τ*_*y *_= 1) yield a high correlation when the extracted features are applied to the test data. The unregularized model extracts spurious features which are very specific to the training data, while the fully regularized model extracts features with high variance, but only moderate correlation. As can be seen in Figure 1, there are several choices of regularization parameters resulting in a model with a high generalization ability but clearly, choosing the gene expression regularization parameter larger than the copy number regularization parameter does not give generalizable features.

To estimate the generalization ability that could be expected for features extracted from paired data without any truly related components, we permute the samples in the gene expression matrix of the tuning data. We then run the 3-fold cross-validation to estimate the generalization ability of the features extracted with the regularization parameters fixed to the optimal values determined above. The mean value ± SD for  across 50 instances with permuted data is  = 0.138 ± 0.074, indicating that the extracted features in this case are very specific to the training set which they were extracted from, and not generalizable. Comparing  to , we conclude that the first feature pair extracted from the original data is indeed likely to represent a true linear relationship between the gene expression and copy number data.

Since the regularization parameters are determined based only on the first pair of extracted features, we estimate the generalization ability also for the second pair of features, with the regularization parameters fixed at *τ*_*x *_= 0.9, *τ*_*y *_= 0.3. The same 3-fold cross-validation strategy is applied to the tuning data, giving  = 0.574. The corresponding value for permuted data (mean ± SD) is  = 0.128 ± 0.059. Hence, also the second pair of features from the regularized dual CCA can be expected to encode a true linear relationship between the two variable sets.

For the PCA+CCA, we extract twelve principal components, independently from each variable set, to use as variables in a classical CCA. This choice is motivated by an intention to keep the number of variables for the CCA low, while still extracting enough information from each of the variable sets. The first twelve principal components explain 52% of the variance in the copy number data set, and 58% of the variance in the gene expression data set, and the scree plots are almost flat (data not shown), indicating that the rest of the components mostly contain noise. Applying the 3-fold cross-validation strategy to test the generalization ability of the first two pairs of PCA+CCA features returns the estimates  = 0.874 and  = 0.743 of the test set canonical correlations. The corresponding values across 50 instances with permuted data matrix (mean ± SD) are  = 0.126 ± 0.063 and  = 0.130 ± 0.056. Hence, we expect also the first two PCA+CCA feature pairs to encode true linear relationships.

### Relevance of the variables

**Figure 2:**
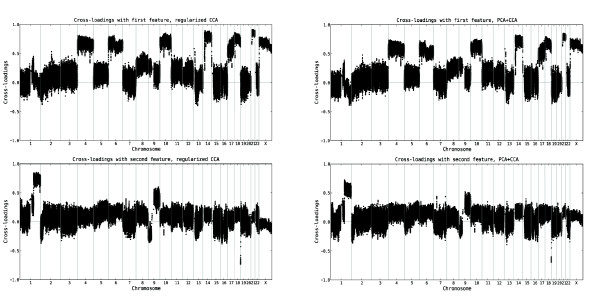
**Relevance of copy number variables to the top CCA features from the leukemia data set**. The cross-loadings, indicating the relevance of each copy number variable to the first two features extracted using regularized dual CCA, with regularization parameters *τ*_*x *_= 0.9, *τ*_*y *_= 0.3 (left panel), and PCA+CCA (right panel). The upper panels show the cross-loadings with the first feature, and the lower panels show the cross-loadings with the second feature. The variables are ordered along the genome and the vertical lines mark the chromosome boundaries. The cross-loadings for a copy number variable measure the degree of linear relationship between the log_2 _ratio of that variable and the respective gene expression features. Hence, a higher (in absolute value) cross-loading indicates a stronger correlation between the variable and the gene expression feature. The first feature is strongly related to copy number alterations affecting chromosomes 4, 6, 8, 10, 14, 17, 18, 21 and X, while the second feature is strongly related to copy number alterations on chromosome 1 and 19, oppositely directed.

**Figure 3:**
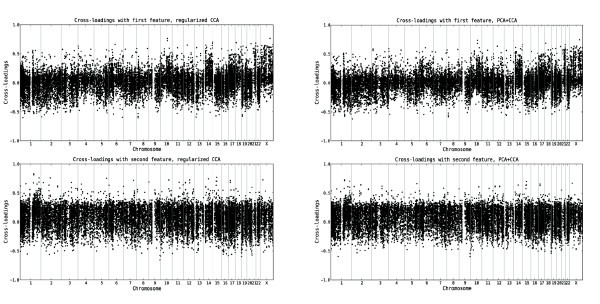
**Relevance of gene expression variables to the top CCA features from the leukemia data set**. The cross-loadings, indicating the relevance of each gene expression variable to the first two features extracted using regularized dual CCA, with regularization parameters *τ*_*x*_= 0.9, *τ*_*y *_= 0.3 (left panel), and PCA+CCA (right panel). The upper panels show the cross-loadings with the first feature, and the lower panels show the cross-loadings with the second feature. The variables are ordered along the genome and the vertical lines mark the chromosome boundaries. The cross-loadings for a gene expression variable measure the degree of linear relationship between the log_2 _expression value of that variable and the respective copy number features. Hence, a higher (in absolute value) cross-loading indicates a stronger correlation between the variable and the copy number feature.

**Figure 4:**
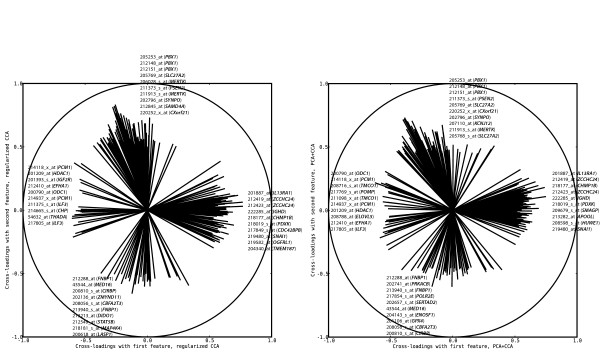
**Relevance of selected gene expression probe sets to the top CCA features**. Graphical representation of the cross-loadings, indicating the relevance of the most important gene expression probe set to the first two features extracted using regularized dual CCA, with regularization parameters *τ*_*x *_= 0.9, *τ*_*y *_= 0.3 (left panel), and PCA+CCA (right panel). The value on the horizontal axis indicates the cross-loading with the first feature, and the value on the vertical axis indicates the cross-loading with the second feature. The circle has radius 1, indicating perfect correlation between the expression of a probe set and a copy number feature. The lines show the cross-loadings for the probe sets with the highest relevance to each of the two features, and the genes corresponding to the ten probe sets with highest positive and negative correlation with each feature, respectively, are given. The overall highest correlation is found with regularized dual CCA (left panel), between a probe set corresponding to the *PBX1 *gene and the second feature.

Given that the first two pairs of features from both regularized dual CCA and PCA+CCA are expected to encode true linear relationships between the copy number and gene expression variables, we interpret the biological content in these by computing the cross-loadings for each variable with the extracted features, based on the tuning samples. The cross-loadings for the copy number and gene expression variables, ordered along the genome, are shown in Figures 2 and 3, respectively. A visualization of the gene expression probe sets which are most relevant (i.e. have the highest cross-loadings) to the two extracted features are shown in Figure 4. Furthermore, lists of the 150 gene expression probe sets showing the highest cross-loadings with each of the two features are available in Additional file 2 and Additional file 3. As can be seen in Figures 2, 3 and 4, many of the most relevant genes (although not all) as well as the overall copy number profiles are shared between the features extracted using the two methods. This indicates a certain robustness of the underlying patterns and strengthens the interpretation that the results are biologically relevant. Indeed, of the 150 probe sets found to have the highest correlation with the first extracted feature pair, 128 are identical for the two methods. For the second feature pair, 116 of the 150 most relevant probe sets are identical for both methods. Apparently, the features showing the strongest linear relationship are characterized mainly by whole chromosome copy number alterations affecting chromosomes 4, 6, 8, 10, 14, 17, 18, 21 and X, and expression changes for genes such as *IL13RA1*, *ZCCHC24 *and *IGHD*. The second pair of features is associated with a large copy number alteration on chromosome 1 and a small change on chromosome 19, oppositely directed. Among the genes with highest cross-loadings with the second feature we note *PBX1*, *SLC27A2 *and *PSEN2*.

### Canonical correlations and sample representations

So far, we have used only the tuning set to determine the optimal regularization parameters and extract two pairs of features from the variable sets. To determine whether there are specific subgroups of the patients that are singled out by the extracted features, and to assess the generalization ability to unseen data, we visualize the samples from the tuning set and the validation set by their coordinates in the extracted features.

**Figure 5:**
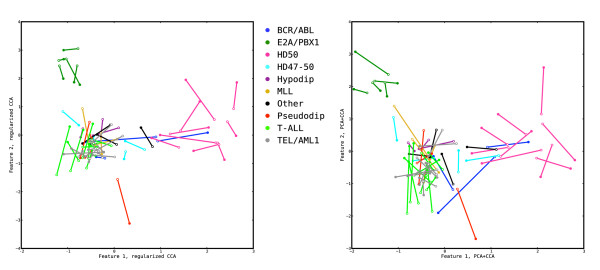
**The extracted features characterize well-known leukemia subtypes**. Representation of the samples from the validation set by their coordinates in the first two pairs of features extracted from the tuning set using regularized dual CCA, with regularization parameters *τ*_*x*_= 0.9, *τ*_*y *_= 0.3 (left panel), and PCA+CCA (right panel). We show the representations with respect to both the copy number features and the gene expression features in a superimposed way, where each sample is represented by two markers. The filled markers represent the coordinates in the features extracted from the copy number variables, and the open markers represent coordinates in the features extracted from the gene expression variables. Samples with different leukemia subtypes are shown with different colors. The first feature pair distinguishes the HD50 group from the rest, while the second feature pair represents the characteristics of the samples from the E2A/PBX1 group.

Application of the features extracted by dual regularized CCA to the tuning data set results in highly correlated sample representations from the copy number and gene expression data. In fact, the correlation between the representations of the samples by the first feature pair is  = 1.000, and for the second feature pair it is  = 1.000. Nevertheless, based on the cross-validation we expect these features to have a high generalization ability, and when applied to the validation data set the canonical correlations are indeed  = 0.905 and  = 0.787 for the two feature pairs, respectively. The canonical correlations for the tuning and validation sets for all methods are given in Table 1. The low-dimensional representations of the samples from the validation and tuning set, respectively, are shown in Figure 5 and Additional file 1: Supplementary Figure 1. In these figures, each sample is represented by two points, joined by a line segment [31]. One point represents the sample by its value on the two gene expression features, and the other point represents the sample by its value on the two copy number features. A high canonical correlation thus implies that the two points corresponding to a sample are very close to each other. This is exactly what we see for the representation of the tuning set (left panel in Additional file 1: Supplementary Figure 1), whereas the lower canonical correlations observed for the validation set imply larger distances between the two points for each sample. In both the tuning and the validation set, the first pair of features (horizontal axis) singles out one group of samples. Applying the subtype information reveals that it consists mainly of patients from the HD50 subgroup. Similarly, the second pair of features (vertical axis) distinguishes one, apparently homogeneous, group of samples from the rest and applying the subtype information this group is identified as the patients with the *E2A/PBX1 *fusion gene. The redundancy coefficients for the tuning and validation sets are shown in Tables 2 and 3, where we see that the fraction of the variance in the copy number variables shared by the extracted gene expression features (*R*_*x*|*y*_) is much larger than the fraction of the variance in the gene expression variables shared by the extracted copy number features (*R*_*y*|*x*_). This may indicate that copy number changes are often related to changes in gene expression, captured by the extracted features, while changes in gene expression may have many other causes which are not encoded by the extracted copy number features. For the tuning set, the redundancy coefficients *R*_*y*|*y *_and *R*_*x*|*x *_are very similar to *R*_*y*|*x *_and *R*_*x*|*y*_, respectively. This is due to the high correlation between the extracted gene expression and copy number features, implying that each extracted gene expression feature encodes almost the same information as the corresponding copy number feature.

**Table 1:** Canonical correlations for tuning and validation data.

Method	Canonical correlations			
				

Optimally regularized dual CCA	1.000	1.000	0.905	0.787

Fully regularized dual CCA	0.531	0.507	0.340	0.212

Non-regularized dual CCA	1.000	1.000	0.187	0.295

PCA+CCA	0.925	0.770	0.878	0.657

PCA	0.112	0.117	0.0004	0.080

*Sparse CCA [21]	0.886	0.513	0.820	0.185

*Optimally regularized dual CCA	1.000	1.000	0.910	0.800

The canonical correlations for methods indicated by * are calculated using a subset of the gene expression and copy number variables.

Canonical correlations for the tuning and validation data set, for the different methods applied in this paper.

**Table 2:** Redundancy coefficients for tuning data.

Method	Redundancy coefficients			
	***R*_*x*|*y*_**	***R*_*y*|*x*_**	***R*_*x*|*x*_**	***R*_*y*|*y*_**

Optimally regularized dual CCA	(0.168, 0.038)	(0.030, 0.035)	(0.168, 0.038)	(0.030, 0.035)

Fully regularized dual CCA	(0.051, 0.008)	(0.029, 0.050)	(0.199, 0.094)	(0.237, 0.278)

Non-regularized dual CCA	(0.004, 0.005)	(0.009, 0.004)	(0.004, 0.005)	(0.009, 0.004)

PCA+CCA	(0.131, 0.028)	(0.026, 0.031)	(0.153, 0.043)	(0.030, 0.049)

PCA	(0.008, 0.021)	(0.023, 0.011)	(0.202, 0.065)	(0.291, 0.053)

*Sparse CCA [21]	(0.126, 0.018)	(0.026, 0.040)	(0.172, 0.093)	(0.040, 0.253)

*Optimally regularized dual CCA	(0.153, 0.038)	(0.031, 0.038)	(0.154, 0.038)	(0.031, 0.038)

Redundancy coefficients for the tuning data set, for the different methods applied in this paper. The redundancy coefficients for methods indicated by * are calculated using a subset of the gene expression and copy number variables.

**Table 3:** Redundancy coefficients for validation data.

Method	Redundancy coefficients			
	***R*_*x*|*y*_**	***R*_*y*|*x*_**	***R*_*x*|*x*_**	***R*_*y*|*y*_**

Optimally regularized dual CCA	(0.113, 0.034)	(0.033, 0.027)	(0.145, 0.053)	(0.042, 0.064)

Fully regularized dual CCA	(0.031, 0.015)	(0.027, 0.028)	(0.175, 0.086)	(0.233, 0.268)

Non-regularized dual CCA	(0.027, 0.027)	(0.023, 0.012)	(0.077, 0.048)	(0.057, 0.020)

PCA+CCA	(0.100, 0.033)	(0.032, 0.031)	(0.118, 0.048)	(0.037, 0.078)

PCA	(0.012, 0.018)	(0.026, 0.022)	(0.179, 0.068)	(0.281, 0.069)

*Sparse CCA [21]	(0.092, 0.014)	(0.033, 0.023)	(0.142, 0.087)	(0.051, 0.252)

*Optimally regularized dual CCA	(0.107, 0.036)	(0.034, 0.029)	(0.131, 0.054)	(0.042, 0.062)

Redundancy coefficients for the validation data set, for the different methods applied in this paper. The redundancy coefficients for methods indicated by * are calculated using a subset of the gene expression and copy number variables.

With PCA+CCA, the canonical correlations for the first two component pairs on the tuning data set are  = 0.925 and  = 0.770, while the canonical correlations for the validation data set are  = 0.878 and  = 0.657, respectively. The canonical correlations for the tuning set are considerably lower than those from the regularized dual CCA, which is an indication of the lower flexibility in PCA+CCA. Indeed, while the regularized dual CCA is free to assign weights to the variables independently, when CCA is applied after PCA each principal component receives a weight. Highly correlated variables are collected with similar weights into the same principal component, and consequently receive a similar total weight after PCA+CCA. However, the canonical correlations for the validation set are similar for the two methods. The high canonical correlations for the validation set show that the features have a high generalization ability to unseen data. The representations of the samples of the validation and tuning sets by the first two pairs of PCA+CCA features are shown in the right panels of Figure 5 and Additional file 1: Supplementary Figure 1, respectively. The group characterized by the first pair of features consists mainly of HD50 patients, as with regularized dual CCA. Notably, the TEL/AML1 sample, located very close to the HD50 group in the regularized dual CCA representations (grey symbol, left panel of Additional file 1: Supplementary Figure 1), now appears similar to the HD50 group with respect to the coordinates in the copy number features while there is a large distance to the coordinates in the gene expression features. The second feature again characterizes the E2A/PBX1 group. The redundancy coefficients are shown in Tables 2 and 3. As with regularized CCA, the gene expression features share more variance with the original copy number variables than oppositely.

### The effect of choosing extreme regularization values

**Figure 6:**
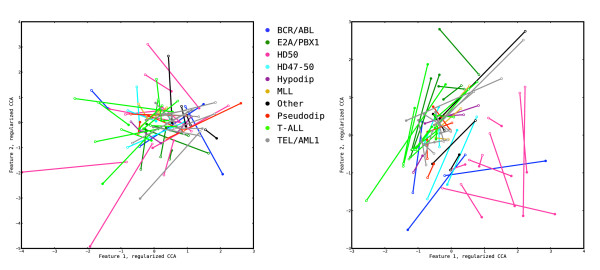
**The effect of choosing extreme regularization values**. Representation of the samples from the validation set by their coordinates in the first two pairs of features extracted from the tuning set using regularized dual CCA, with regularization parameters *τ*_*x *_= 0, *τ*_*y *_= 0 (left panel), and *τ*_*x *_= 1, *τ*_*y *_= 1 (right panel). The filled markers represent the coordinates in the features extracted from the copy number variables, and the open markers represent coordinates in the features extracted from the gene expression variables. Samples with different leukemia subtypes are shown with different colors. Without regularization (left panel) the extracted features, although being highly correlated (see Additional file 1: Supplementary Figure 2), do not encode any biological information and are not generalizable to extract correlated information from the validation set. With maximal regularization (right panel), the agreement between the extracted features is weak, although they contain a large part of the variance in the data sets.

In Figure 6 and Additional file 1: Supplementary Figure 2 we show the effect of choosing *τ*_*x *_= *τ*_*y *_= 0 and *τ*_*x *_= *τ*_*y *_= 1, respectively, on the representation of the validation and tuning samples by their coordinates with respect to the first two pairs of features extracted with regularized dual CCA. In the unregularized case (left panels), although the correlation between the extracted features is very high (the two points for each sample in the tuning data coincide, left panel of Additional file 1: Supplementary Figure 2) the generalization ability of the features is very low, as can be seen by the long distances between points in Figure 6. This indicates that the features do not encode any true biological information. Furthermore, the cross-loadings for all variables with the first two features are very low, hence no variables appear to be strongly related to the extracted features (data not shown).

In the fully regularized, i.e. covariance-maximizing, case (right panels of Figure 6 and Additional file 1: Supplementary Figure 2), the correspondence between the features from the two variable sets is much weaker (there is a long distance between the two points for each sample also in the tuning set) and the biological information is less clear than with the optimal choice of regularization parameters. Studying the redundancy coefficients for this method (see Tables 2 and 3), we conclude that more emphasis is put on extracting features which explain a large part of the variance in the gene expression data set, compared to the optimally regularized dual CCA and the PCA+CCA, which put more focus on the correlation structure. The resulting canonical correlations for the first two feature pairs are  = 0.531 and  = 0.507 for the tuning data and  = 0.340 and  = 0.212 for the validation data. Hence, in terms of the canonical correlations in the validation set, the features from fully regularized CCA are much less generalizable than those from the optimally regularized dual CCA and PCA+CCA.

### Comparison to a sparse covariance-maximizing method

To further evaluate the regularized dual CCA and contrast its findings to those from a sparse covariance-maximizing method, we compare it to the diagonal penalized CCA described by Witten *et al*. [21] using the R package implemented by the authors. This method is fully regularized, hence covariance-maximizing. For computational feasibility, the comparison was performed on a subset of the data, including only those SNPs that are situated within the boundaries of a gene from the gene expression data set. The genes not harboring any SNPs were also removed, leaving a total of 57,814 copy number variables and 11,685 gene expression probe sets. The optimal regularization parameters for the regularized dual CCA are in this case *τ*_*x *_= 0.75, *τ*_*y *_= 0.3, reflecting the considerable reduction in the number of variables, in particular for the copy number variable set. As before, we extract two pairs of features. The optimal degree of sparsity for the sparse CCA is estimated using the permutation routine implemented in the R package. In this package, the sparsity parameters are estimated based on the first feature pair.

**Figure 7:**
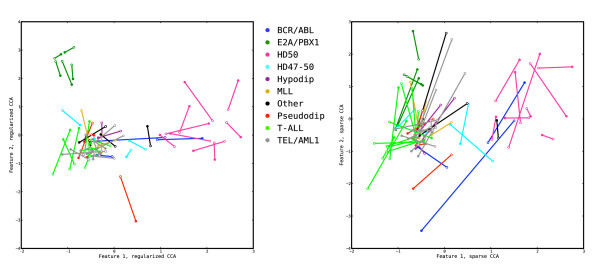
**Comparison of sample representations with regularized dual CCA and sparse CCA**. Representation of the samples from the validation set by their coordinates in the first two pairs of features extracted from the tuning set using regularized dual CCA (left panel, *τ*_*x *_= 0.75, *τ*_*y *_= 0.3) and the sparse CCA proposed by Witten *et al*. [21] (right panel). The filled markers represent the coordinates in the features extracted from the copy number variables, and the open markers represent coordinates in the features extracted from the gene expression variables. Samples with different leukemia subtypes are shown with different colors. For computational reasons, the comparison is performed using a subset of the copy number and gene expression variables. The features extracted using regularized dual CCA have a higher correlation, as shown by the shorter distance between the points corresponding to each sample in the left panel. Moreover, the E2A/PBX1 group is more easily discernible using regularized dual CCA.

**Figure 8:**
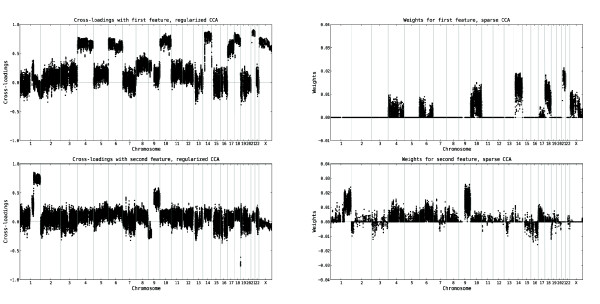
**Relevance of copy number variables to features extracted using regularized dual CCA and sparse CCA**. The relevance of the copy number variables to the extracted features. For regularized dual CCA, the relevances are measured by the cross-loadings with the extracted features (left panel). For sparse CCA, the relevances are measured by the weights of the variables in the extracted features (right panel). The sparse method includes only a subset of the variables, necessary for explaining the information encoded in each feature. The regularized dual CCA (left panel) provides a relevance ranking of all variables. Note that only a subset of the original variable set was used in this analysis.

**Figure 9:**
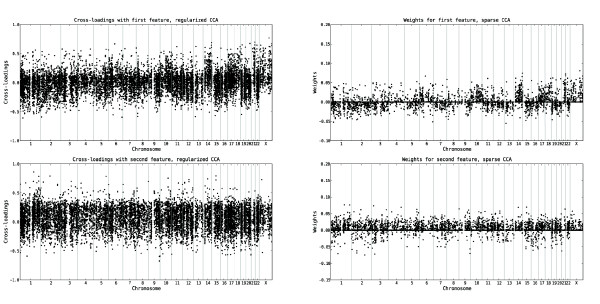
**Relevance of gene expression probe sets to features extracted using regularized dual CCA and sparse CCA**. The relevance of the gene expression probe sets to the extracted features. For regularized dual CCA, the relevances are measured by the cross-loadings with the extracted features (left panel). For sparse CCA, the relevances are measured by the weights of the variables in the extracted features (right panel). The sparse method includes only a subset of the variables, necessary for explaining the information encoded in each feature. The regularized dual CCA (left panel) provides a relevance ranking of all variables. Note that only a subset of the original variable set was used in this analysis.

The regularized dual CCA described in this paper and the sparse CCA of Witten *et al*. [21] have different objectives, which is clearly seen in the results. The regularized dual CCA puts more emphasis on maximizing the correlation of the extracted features, which becomes apparent when studying the samples from the tuning set (Additional file 1: Supplementary Figure 3). In the sparse CCA, since the covariance matrices of the variables are replaced by identity matrices, the covariance of the extracted features is maximized instead. This means that the canonical correlations are expected to be lower, which is also seen by the longer distances between the points for each sample in the right panel of Figure 7. We also note that the E2A/PBX1 group is less discernible with the sparse CCA features, especially with respect to the gene expression features (open markers, right panel of Figure 7). Furthermore, while the regularized dual CCA evaluates the relevance of all variables using the cross-loadings with the extracted features, thereby creating relevance ranking lists for the two variable sets, the sparse CCA attempts to find a suitable subset of the variables, necessary for explaining each feature. This is shown in the right panel of Figure 8, where many variables which are not needed to explain the first component receive a zero weight. The copy number variables which receive a non-zero weight in the first sparse CCA component all have high cross-loadings in the dual regularized CCA. Figure 9 shows the cross-loadings and weights, respectively, for the gene expression variables in the features extracted with the regularized dual CCA and the sparse CCA.

The canonical correlations for the first two feature pairs from the sparse CCA are  = 0.886 and  = 0.513 for the tuning data and  = 0.820 and  = 0.185 for the validation data, respectively. For the regularized dual CCA we get the canonical correlations  = 1.000 and  = 1.000 for the tuning data and  = 0.910 and  = 0.800 for the validation data. From the redundancy coefficients, we note that the sparse CCA features encode slightly more of the variance in the associated data set, but a lower fraction of the variance in the other data set compared to the regularized dual CCA features.

### Analyzing the data sets separately using PCA

**Figure 10:**
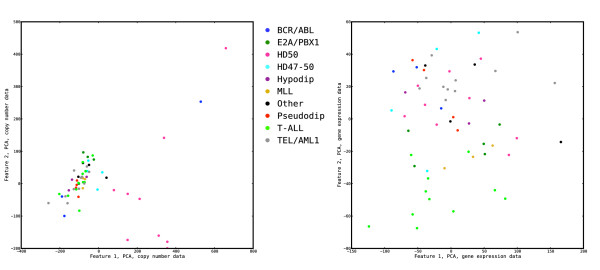
**Analysis of the variable sets separately using PCA**. Representation of the samples from the validation set by the projection onto the first two principal components from the copy number data (left panel) and gene expression data (right panel), extracted from the tuning set. Samples with different leukemia subtypes are shown with different colors. The E2A/PBX1 subgroup, well separated from the rest using the correlation-maximizing methods (Figure 5), is not emerging as a deviating group when the data sets are analyzed individually with PCA. The T-ALL subgroup (light green) has a specific gene expression pattern, and is deviating from the rest using PCA on only the gene expression data (right panel). However, since these subjects do not have a specific copy number pattern, they are less discernible using CCA.

In this section, we compare the results obtained with the integrative correlation-maximizing methods, i.e. regularized dual CCA and PCA+CCA, to those obtained by applying only PCA to each variable set separately. As before, we extract the first two principal components from the tuning set and apply them to the validation set. Figure 10 and Additional file 1: Supplementary Figure 4 show the projection of the validation and tuning samples onto the first two principal components from the copy number data (left panels) and the gene expression data (right panels). Using only the copy number data (left panels), the HD50 subgroup is distinguishable in the first component. However, no further subtype information is visible with this representation. Using only the gene expression data (right panels), the second feature is mainly characterizing the T-ALL subgroup while there is no clearly interpretable information provided by the first feature. With PCA the features are not extracted to be related to the other data set. This agrees with the low canonical correlations, both for the tuning and validation set (Table 1). The redundancy coefficients *R*_*x*|*y *_and *R*_*y*|*x *_are also very low (see Tables 2 and 3). On the other hand, the features are extracted to share as much variance as possible with the variable set they are extracted from, yielding high values of *R*_*x*|*x *_and *R*_*y*|*y*_. Note that since the principal components are required to be uncorrelated, in this case the sum of the fraction of the total variance of the associated data set shared by the first two features (i.e. *R*_*x*|*x *_and *R*_*y*|*y*_) is lower with PCA than with fully regularized CCA. The fraction of variance shared by the first feature only is higher with PCA than with any of the other methods, as expected.

## Discussion and Conclusions

With the rapid development of new genetic measurement methods, there is an increasing interest in combining several types of genetic markers measured in the same samples. Previously, several multivariate methods have been applied to this type of data. For microarray data these methods are mostly covariance-maximizing [20-23] which facilitates application to large data sets. However, as we have shown in this study covariance-maximizing methods may return features which explain a large part of the variance in the individual data sets but show only moderate correlation. A regularized correlation-maximizing method was applied by González *et al *[19,24], but when the number of variables increases the computational complexity of this method may become prohibitive. In this paper we have discussed two methods for integrating paired genetic data sets comprising a very large number of variables, while putting the main emphasis on the correlation of the extracted features, instead of the covariance. First we presented a ridge regularized CCA which was translated to its dual formulation to permit application to data sets with many variables. Second we demonstrated the applicability of a classical CCA, preceded by separate dimension reduction of the two variable sets by PCA, to extract correlated features from a large data set.

We applied the regularized dual CCA and PCA+CCA to a large paired data set consisting of gene expression and SNP copy number measurements from 173 patients with leukemia. With both regularized dual CCA and PCA+CCA we extracted two pairs of highly correlated features from the gene expression and copy number variable sets. We interpreted the biological content of the features using the cross-loadings of all variables with them. Importantly, we noted that even though the feature vectors extracted with the two methods, and hence the representation of the samples, were quite different the interpretation in terms of the cross-loadings of all variables with the extracted features showed a high degree of similarity.

Furthermore, we represented the samples, both from the tuning set used to extract the features and from a validation set, by their coordinates in the CCA features and extracted groups of patients with characteristic gene expression and copy number profiles. The first feature pair, characterized by copy number alterations on chromosomes 4, 6, 8, 10, 14, 17, 18, 21 and X and expression changes for genes such as *ZCCHC24*, *IL13RA1 *and *IGHD*, distinguishes a group of patients from the rest. This group consists mainly of patients from the HD50 subgroup. Notably, the copy number effects dominating this feature agree well with those reported in a large study of patients with the HD50 subtype [32]. Furthermore, in a previous study of the gene expression component of the data set we have used [33], probe sets corresponding to the *ZCCHC24*, *APOOL*, *HUWE1 *and *SMAGP *genes were found among the top 100 probe sets characterizing the HD50 subtype.

The second pair of features is characterized by a large copy number alteration on chromosome 1, and a small, oppositely directed, alteration on chromosome 19. The gene which is most highly related to the extracted copy number pattern is *PBX1*, followed by e.g. *SLC27A2 *and *PSEN2*. This feature pair (together with the first pair) clearly separates one group of samples from the rest. Applying the subtype information, this group is seen to consist exclusively of samples from the E2A/PBX1 group. This subtype is indeed characterized by a translocation between chromosomes 1 and 19 which gives rise to the *E2A/PBX1 *fusion gene. While balanced translocations cannot be detected by SNP-arrays, the 1;19 translocation is in most cases (and in the present data set, all cases) present as an unbalanced translocation, resulting in gain of 1q and loss of 19p material. Furthermore, many of the genes showing high cross-loadings with the second feature (see Figure 4) have been shown to be characteristic to *E2A/PBX1*-positive ALLs [33,34]. The higher subtype specificity of the genes associated with the second feature is consistent with the clear separation of the samples with the *E2A/PBX1*-positive subtype from the rest in Figure 5. Notably, without prior knowledge of subgroups in the data set, and without pre-selection of variables, we have used the correlation structure between copy number alterations and gene expression changes to extract two well-known subtypes having specific gene expression as well as copy number profiles.

The representations of the samples by the values of the extracted features (Figure 5) show the qualitative similarities and differences between the regularized dual CCA and PCA+CCA. As shown in Figures 2, 3 and 4, the cross-loadings for the variables are similar for the regularized dual CCA and PCA+CCA, which implies that the extracted features encode the same underlying biological information. This can also be seen by comparing the sample representations from the two methods (compare the two panels of Figure 5), where the overall distributions of the samples are similar. Despite this, the actual weights of the variables in the features from the two methods are quite different due to the higher flexibility in choosing the weights in the regularized dual CCA as compared to PCA+CCA. For example, although one TEL/AML1 sample is similar to the HD50 samples with regards to the copy number profile and not as similar with regards to the gene expression profile, the regularized dual CCA anyway succeeds in finding a pair of features which has a high correlation, due to the high flexibility in assigning weights to the variables. Unlike in PCA+CCA, correlated variables do not necessarily receive similar weights, and it is thus possible to choose a suitable subset from a set of correlated variables to increase the correlation, at the expense of a lower variance of the extracted features.

Previously, gene expression profiling has been applied to extract subgroups of leukemia samples (see e.g. [33]). In such studies the T-ALL subgroup often emerges as having a different gene expression pattern than the other subtypes. Since the methods applied in our study focus on correlations between gene expression and copy number changes and patients from the T-ALL subgroup do not have a characteristic copy number profile, they will not emerge as a deviating group in our analysis. On the other hand the characteristics of the HD50 and E2A/PBX1 subgroups are considerably weaker if we study gene expression or copy number data separately using PCA. Since both of these groups have strong copy number profiles as well as specific changes in the expression of characteristic sets of genes, integrative analysis of these two variable sets allows for them to be extracted. We point out the advantage of analyzing a data set with several different methods, since they may very well yield different biological information. We conclude that CCA, either regularized and translated to its dual formulation or combined with PCA, can be applied to high-dimensional paired data sets to allow for efficient exploratory integrative analysis without using prior knowledge or pre-selection of variables. As such, it is a valuable tool for generating hypotheses from high-dimensional data sets. Furthermore, while we in this study only have searched for linear relationships between the copy number and gene expression variable sets the dual formulation of CCA can be generalized to extract nonlinear relationships by the kernel trick. Compared to previously proposed sparse integrative methods, where computational problems often lead to a focus on maximizing the covariance instead of a penalized correlation, the methods proposed in this paper may be valuable for finding closely related patterns which do not necessarily correspond to a large part of the variance in the data set.

## Methods

### Data and pre-processing

The data set used in this study was generated and first described by Mullighan *et al *[35]. It includes 173 childhood acute lymphoblastic leukemia (ALL) patients, for which both gene expression and SNP copy numbers were measured. The leukemias were classified, using lineage information (B-cells or T-cells), karyotyping, reverse transcriptase PCR and fluorescence *in situ *hybridization, into the following subtypes: T-ALL, hyperdiploidy (more than 50 chromosomes on karyotyping, abbreviated here as HD50), *E2A/PBX1*-positive, *TEL/AML1*-positive, *BCR/ABL1*-positive, *MLL*-rearranged, low hyperdiploidy (47-50 chromosomes, abbreviated here as HD47-50), hypodiploidy, pseudodiploidy or other [35].

The gene expression data set was generated using Affymetrix HG-U133A arrays, providing mRNA levels of more than 18,000 transcripts. The copy number data set was generated using Affymetrix Human Mapping 250 K Sty SNP-arrays which give copy number and genotype information for more than 230,000 SNPs.

The CEL-files containing raw intensity signals from the Affymetrix HG-U133A arrays and the CEL- and CHP-files containing raw intensity signals and genotype information from the Affymetrix Human Mapping 250 K Sty arrays were downloaded from http://hospital.stjude.org/forms/genome-download/request/. The raw intensity signals from the gene expression arrays were normalized using gcRMA [36] which is a part of the Bioconductor [37] package for R (R Development Core Team, 2008). To avoid difficulties with interpretation, 2,234 probes corresponding to more than one genomic location were removed from the analysis.

The CEL- and CHP-files from the SNP-arrays were imported into dChip [38], where the raw signals were normalized to a baseline level using an invariant set of probes and the "expression level" of each SNP was calculated using model based expression with perfect match/mismatch difference. A reference copy number for each SNP was calculated from all samples by trimming the 25% extreme values in both ends. The copy number changes were calculated using median smoothing with a 10 SNP window and the dChip option "scale copy number mode to 2 copy". Copy number calculations were performed in small batches based on the creation dates of the CEL-files to eliminate strong batch-specific effects observed if all data were analyzed together.

In all analyses, we use the log_2 _ratios from the copy number data set and the log_2_-transformed gene expression values for the gene expression probe sets. Before entered into the canonical correlation analysis, each variable is mean-centered and standardized to unit variance across the samples used for feature extraction. Whenever a training set and a test set are used, both sets are standardized using the mean value and standard deviation from the training set. The final data set, to which we apply the integrative methods, consist of 20,021 gene expression probe sets and 238,304 copy number variables.

### Canonical Correlation Analysis

Canonical Correlation Analysis (CCA [5]) is a generalization of multiple linear regression to the case of several response variables. It is applicable to paired data sets, consisting of two sets of variables measured on the same samples and represented by two matrices **X **∈ ℝ^*n *× *N *^and **Y **∈ ℝ^*m *× *N*^, where each column in **X **and **Y **corresponds to one of the *N *samples, and each row represents one variable. We let **X **denote the copy number data matrix and **Y **the gene expression data matrix, and assume that each variable is centered to have zero mean across the samples. Furthermore, we let **C**_*xx *_= **XX**^*T *^and **C**_*yy *_= **YY**^*T *^denote the (scaled) sample covariance matrices for the two variable sets, and **C**_*xy *_= **XY**^*T *^the (similarly scaled) sample cross-covariance matrix.

The objective of CCA is to extract, from the two variable sets, latent features which are most highly correlated. The latent features returned by CCA are linear combinations of the measured variables. More formally, we are searching for weight vectors **w**_*x *_∈ ℝ^*n *^and **w**_*y *_∈ ℝ^*m *^such that the empirical correlation between the respective projections onto these weight vectors, i.e. between **X**^*T *^**w**_*x *_and **Y**^*T *^**w**_*y*_, is maximized. Hence, we are seeking to maximize(1)

Subsequent features can be extracted by maximizing the correlation for projections onto weight vectors uncorrelated to those extracted previously. This formulation of CCA also shows that it is completely symmetric in the two variable sets. If **C**_*xx *_and **C**_*yy *_are invertible, the weight vectors maximizing (1) can be found by solving the eigenvalue problems(2)

### Application of CCA to high-dimensional data sets

Since the classical formulation of CCA requires that the sample covariance matrices **C**_*xx *_and **C**_*yy *_are invertible, it is not uniquely solvable when the number of variables exceeds the number of samples, and it can be severely confounded by collinearities among the variables. Both of these characteristics are common in microarray data sets. In these cases we need to reduce the flexibility of the CCA, thereby increasing the robustness of the feature extraction. We address this issue in two different ways, first by regularizing the CCA explicitly and second by reducing the dimensionality of the data using PCA before applying classical CCA.

#### Regularized dual CCA

We regularize the classical CCA by adding a ridge penalty to the covariance matrices. This is a commonly used regularization method (applied with various choices of regularization degrees e.g. in [19-22,30]) that was introduced in the CCA framework by Vinod [7]. Hence, we are seeking **w**_*x *_and **w**_*y *_to maximize the penalized correlation

where *N *is the number of samples in the data set. We introduce the *N *into the expression to render the two terms in each parentheses in the denominator of approximately equal magnitude, at least in the case where the variables within each set are uncorrelated and of unit variance (in which case both **C**_*xx *_and **C**_*yy *_are approximately *N I*). The regularization parameters *τ*_*x *_and *τ*_*y *_parametrize a whole family of methods, ranging from classical CCA (*τ*_*x *_= *τ*_*y *_= 0) which extracts maximally correlated features, to PLS (*τ*_*x *_= *τ*_*y *_= 1) where the extracted features are those showing maximal covariance. The higher value of *τ*_*x *_and *τ*_*y *_that are used, the more focus is put on the variance of the extracted features. Therefore, using regularized CCA implies a trade-off between explaining the variable sets individually and explaining their correlation structure. Introducing the regularization makes it possible to solve the optimization problem even when the number of variables exceeds the number of samples. Maximal regularization, on the other hand, is not necessarily desirable since in this case relevant features with high correlation between the variable sets may be drowned by features with high variance and only moderate correlation.

In cases where the number of variables is very large, it can be a computational advantage to solve the regularized CCA problem in its dual formulation [39]. The dual formulation of CCA can also be used to generalize the method to find nonlinear relationships, by the kernel trick [40-42]. Working in the dual formulation, instead of performing calculations involving the sample covariance matrices **C**_*xx *_= **XX**^*T *^and **C**_*yy *_= **YY**^*T *^we work with the matrices **X**^*T *^**X **and **Y**^*T *^**Y **which are of dimension *N *× *N *and hence much smaller. By expressing the weight vectors **w**_*x *_and **w**_*y *_in terms of the sample matrices as **w**_*x *_= **X***α*_*x *_and **w**_*y *_= **Y***α*_*y*_, substituting into (3) and noting that the resulting expression is invariant to scaling of *α*_*x *_and *α*_*y*_, we obtain the dual formulation of regularized CCA as the optimization problem

Using the notation *K*_*X *_= **X**^*T*^**X **and *K*_*Y *_= **Y**^*T*^**Y**, this can be restated as the generalized eigenvalue problem

which is solved to return *α*_*x *_and *α*_*y*_. The regularized CCA features are then calculated as **w**_*x *_= **X***α*_*x*_, **w**_*y *_= **Y***α*_*y*_. In general, as discussed e.g. in [25], unregularized dual CCA (*τ*_*x *_= *τ*_*y *_= 0) is likely to overfit the data when the number of variables is very large, and should therefore be interpreted with great caution.

In order for the extracted features to be of practical use, they should encode characteristics which are in some sense general to the population to which the considered data set belongs. To achieve this goal we determine the optimal regularization parameters by cross-validation. First, the data set is divided into a tuning set, consisting of two thirds of the samples, and a validation set, consisting of the remaining one third of the samples. The tuning set is used to determine regularization parameters and extract features, and the validation set is used only for visualization and assessment of the generalization ability of the extracted features. To choose the optimal regularization parameters *τ*_*x *_and *τ*_*y *_based on the tuning set, we employ a 3-fold cross-validation technique, with the aim of finding the pair of regularization parameters giving the highest generalization ability of the first extracted pair of features to unseen data. Leave-one-out cross-validation to find the optimal regularization parameter in CCA was proposed by Leurgans *et al*. [30], and thereafter applied by González *et al*. [24]. More recently, González *et al*. [19] applied 5-fold cross-validation for the same purpose. The 3-fold split of the data is chosen to avoid overestimating the generalization ability of the extracted features, which could be the case if the leave-one-out procedure is employed. Moreover, a small fold is chosen to reduce the computational time needed for the parameter selection, and to provide a large enough basis for estimating the canonical correlations in the test sets. Hence, we divide the samples of the tuning set into three groups, with approximately the same distribution of patients from the different subtypes. Two of the groups (the training set) are then used to extract a pair of latent features with the highest value of the penalized correlation (3). The third group of samples (the test set) is then projected onto this pair of features and the empirical correlation for the projected test samples is calculated. This is repeated three times, until all samples have been used once as a test sample, and thereafter we average the three empirical correlations. Letting  and  denote the maximizers of (3) when group *i *is removed from the training set and with **X**^*i *^and **Y**^*i *^denoting the parts of **X **and **Y **corresponding to group *i*, we let (**X**^*i*^)^*T *^ and (**Y**^*i*^)^*T *^ be the projection of the test set (group *i*) onto the extracted features. The estimated test set canonical correlation is then defined as

The same objective function was used by Parkhomenko *et al*. [22] to estimate sparsity parameters. The average is taken over the absolute value of the correlations, since there is no canonical way of choosing the sign of the extracted CCA features.

This analysis is performed for values of *τ*_*x *_and *τ*_*y *_on a grid (following e.g. [43]), and the optimal values are considered to be those maximizing the estimated test set correlation . This correlation can be seen as an estimate of the generalization ability of the first extracted canonical feature pair to unseen data. A high value, meaning that the features with the highest penalized correlation in the training set also have a high correlation in the test set, indicates that the features contain in some sense "true" biological information. The possibility of choosing different regularization parameters for the two variable sets allows us to account for the different properties of the two types of data. Because copy number alterations in cancer often affect large genomic regions, many copy number variables will be highly correlated. We anticipate that, due to this high collinearity among the copy number variables, the copy number data will need more regularization than the gene expression data.

Since the determination of the regularization parameters is based only on the optimization of the largest test set canonical correlation, we also estimate the generalization ability of the second pair of extracted features, to determine if this can be expected to represent a true linear relationship between the two variable sets. Furthermore, to determine whether the estimated test set canonical correlation is larger than what could be expected by chance only we permute the samples in the gene expression variable set and thereby obtain a paired data set without any true correlations. We then estimate the largest test set canonical correlation for the features extracted from the permuted data using the optimal regularization parameters determined for the original data. To find regularization parameters which are in some sense optimal to all extracted feature pairs, it is possible to change the objective function to include not only the largest test set canonical correlations, but also the subsequent ones. For the data set used in this study, reformulating the objective function to the mean of the first and second test set canonical correlation only has a marginal effect and does not change the interpretations.

#### PCA+CCA

Another way to overcome the problem with high data dimensionality is to reduce the dimensionality before applying CCA. We use PCA [6] to reduce the dimensionality of each set of variables independently. The extracted principal components are then used as variables in the CCA. This approach is discussed e.g. by Muller [29]. The weights from the PCA and CCA are combined to yield a total weight for each measured variable in each of the extracted CCA features in the following way. Let ∏_*x *_∈ ℝ^*n *× *s *^and ∏_*y *_∈ ℝ^*m *× *s*^, with *s *≪ *N*, denote the matrices of principal components resulting from applying PCA to each data set separately. Projecting **X **and **Y **onto the principal components yields *s*-dimensional representations such that as much as possible of the variance from each variable set is retained. Furthermore, if **X **and **Y **are mean-centered, the same will be true for the projections  and . Now CCA is applied to  and , extracting weight vectors  and  to maximize the empirical correlation between  and . Since the "variables" in  and  are now uncorrelated and much fewer than the samples,  and  can be calculated using (2). Hence, in this stage, each principal component from the two data sets receives a weight. The total weights for the original variables are created as , . This construction implies that two highly correlated variables, which thus receive similar weights in each of the principal components, will have similar weights also in the total weight vectors.

This approach is intuitively appealing in that we first extract the most variable (and hence, in some sense, most informative) features from each data set and then search for the highest correlations between combinations of these. Hence it prevents the CCA from finding high correlations between features of very low variance, which are presumably mostly noise. When using PCA+CCA it is important to be aware that discarding all but the first *s *principal components, while being advantageous for noise reduction, may result in some highly correlated features of low variance being thrown away. With regularized CCA there is a possibility, depending on the choice of regularization parameters, that such features may be found. The features extracted with PCA+CCA undergo the same cross-validation as the regularized dual CCA features to estimate whether the obtained test set canonical correlations are larger than could be expected by chance only.

### Visualization and interpretation of CCA features and sample representations

The weights **w**_*x *_and **w**_*y *_from CCA are sensitive to collinearities among variables, and hence not necessarily a good way of evaluating the individual contribution of each variable to the CCA features. Instead, the *cross-loadings *of the variables can be used to interpret the extracted features [28]. The cross-loading of the *i*'th variable from one of the variable sets with the *j*'th pair of extracted features is defined as the correlation between the variable and the *j*'th extracted feature from the *other *variable set. This means that the cross-loadings indicate the relevance of each variable to each extracted feature pair. Highly correlated variables obtain similar cross-loadings, even though their weights in the extracted features may be different. The relevances of the copy number and gene expression variables are visualized by showing their cross-loadings along the genome, see Figures 2 and 3. To further visualize the cross-loadings of the most relevant gene expression probe sets to the extracted features, as well as the correlation structure between these, the highest cross-loadings with the first two features are shown in the same figure (Figure 4) [19,44].

To visualize the representations of the samples, we use their coordinates in the first two pairs of extracted features and show them in a superimposed way [31]. Using this approach, each sample is represented by two points joined by a line segment, in a two-dimensional space. Each point represents the coordinates of the sample in the features extracted from one of the two variable sets. Since we are mainly interested in the correlation structure, each feature is standardized to unit variance prior to this visualization. Hence, if the correlation between the extracted features from the two data sets is close to one, the two points for each sample will almost coincide. The representations are shown for the samples in the tuning set (Additional file 1: Supplementary Figures 1, 2, 3 and 4) and for the samples in the validation set (Figures 5, 6, 7 and 10).

To obtain an estimate of the fraction of the variance in the original variable sets which is shared by the extracted features, we calculate the redundancy coefficients [28,44]. The redundancy coefficient of the two features extracted from the *x *variable set with respect to the original *y *variables, indicating the fraction of the variance in the original *y*-variables shared by the extracted *x*-features, is denoted by *R*_*y*|*x*_, with similar interpretations of *R*_*x*|*y*_, *R*_*x*|*x *_and *R*_*y*|*y*_. Denoting the cross-loading of *y*-variable *i *with the *j*'th feature from the *x *variable set by *c*_*ij *_for *i *= 1,..., *m *and *j *= 1, 2, the redundancy coefficient with the two features is calculated as

By replacing the cross-loadings of variable *i *with the correlations between the variable and the features extracted from its associated data set, we obtain instead *R*_*y*|*y*_. *R*_*x*|*y *_and *R*_*x*|*x *_are defined accordingly.

## Authors' contributions

CS designed and implemented the computational analysis, participated in the interpretation of the results and drafted the manuscript. HL pre-processed the data, participated in the interpretation of the results and helped drafting the manuscript. TF and MF contributed to the design of the study, the interpretation of the results and revision of the manuscript. All authors read and approved the final manuscript.

## Supplementary Material

Additional file 1 **Additional file**1** contains Supplementary Figures** 1, 2, 3 **and** 4, **which show the representation of the samples from the tuning set by the coordinates in the features extracted by the different methods**. Click here for file

Additional file 2 **Additional file **2** contains the cross-loadings of the 150 gene expression probe sets showing the highest relevance to the two extracted features from regularized dual CCA with regularization parameters *τ*_*x *_= 0.9 and *τ*_*y *_= 0.3.** Click here for file

Additional file 3 **Additional file**3**contains the cross-loadings of the 150 gene expression probe sets showing the highest relevance to the two extracted features from PCA+CCA.** Click here for file
