# Supplementary material for: Integrative analysis of gene expression and copy number alterations using canonical correlation analysis
Source: BMC Bioinformatics. 2010 Apr 15;11:191. doi: 10.1186/1471-2105-11-191 (PMC2873536; doi:10.1186/1471-2105-11-191)
Supplement: Additional file 1 — Additional file1 contains Supplementary Figures 1, 2, 3 and 4, which show the representation of the samples from the tuning set by the coordinates in the features extracted by the different methods. [file 1471-2105-11-191-S1.PDF]

Integrative analysis of gene expression and copy number alterations using canonical correlation analysis

Supplementary Figures

Charlotte Soneson, Henrik Lilljebjörn, Thoas Fioretos, Magnus Fontes

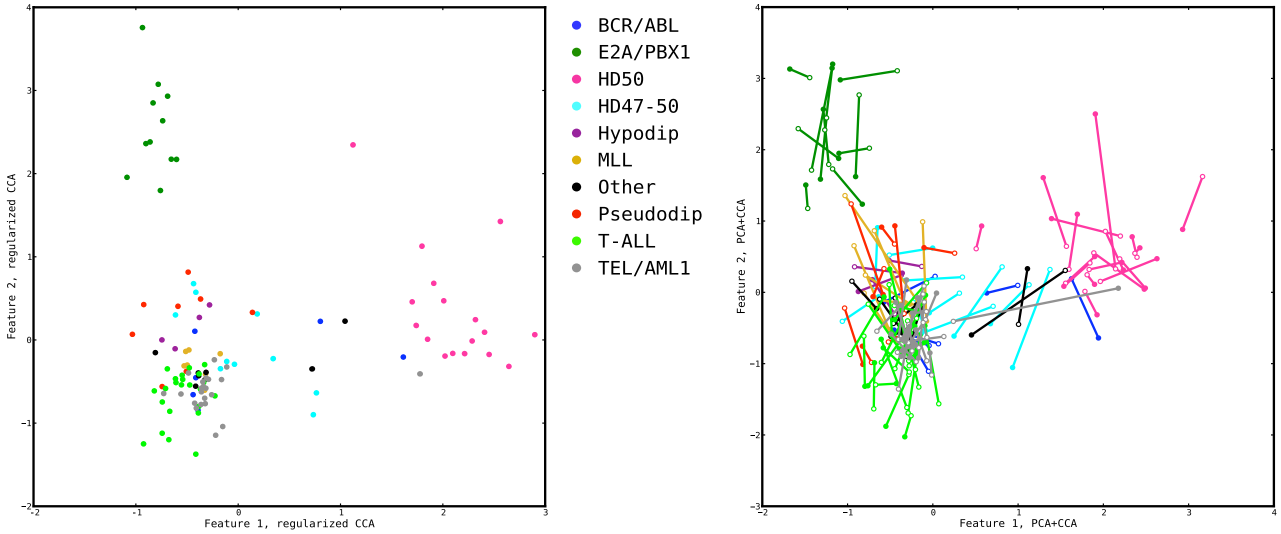

Supplementary Figure 1. *Representation of the samples from the tuning set by their coordinates in the first two pairs of features (extracted from the tuning set) using regularized dual CCA, with regularization parameters* $\tau_x = 0.9$*,* $\tau_y = 0.3$ *(left panel), and PCA+CCA (right panel). We show the representations with respect to both the copy number features and the gene expression features in a superimposed way, where each sample is represented by two markers. The filled markers represent the coordinates in the features extracted from the copy number variables, and the open markers represent coordinates in the features extracted from the gene expression variables. Samples with different leukemia subtypes are shown with different colors. The first feature pair distinguishes the HD50 group from the rest, while the second feature pair represents the characteristics of the samples from the E2A/PBX1 subtype. The high canonical correlation obtained for the tuning samples with regularized dual CCA is apparent in the left panel, where the two points for each sample coincide. Nevertheless, the extracted features have a high generalization ability, as can be seen in the left panel of Figure 5, showing the representation of the validation samples.*

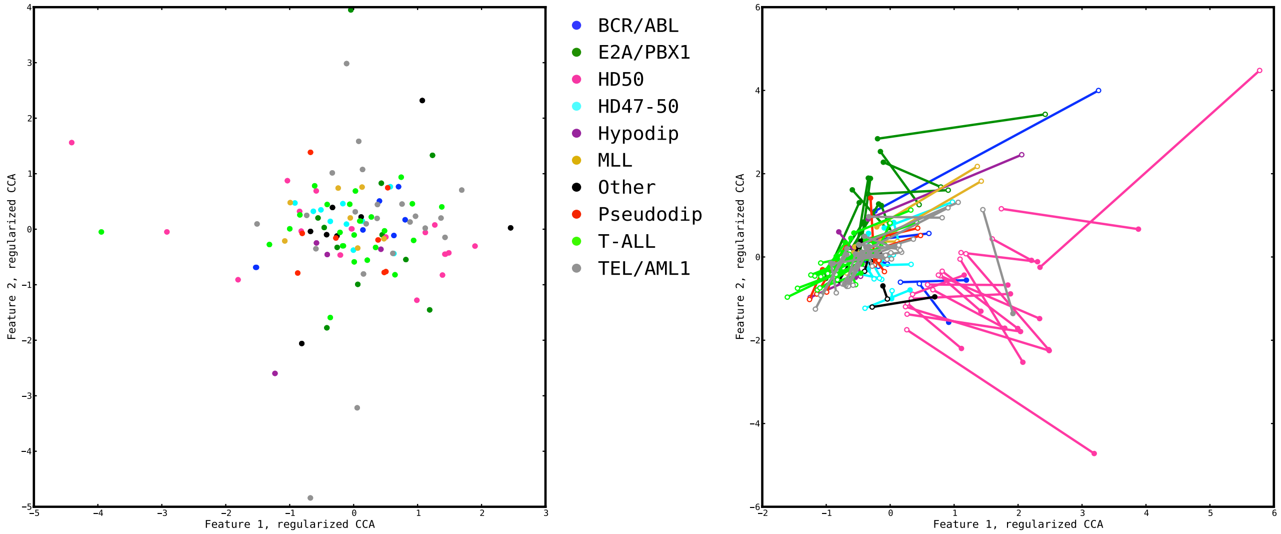

Supplementary Figure 2. *Representation of the samples from the tuning set by their coordinates in the first two pairs of features (extracted from the tuning set) using regularized dual CCA, with regularization parameters* $\tau_x = 0$, $\tau_y = 0$ *(left panel), and* $\tau_x = 1$, $\tau_y = 1$ *(right panel). We show the representations with respect to both the copy number features and the gene expression features in a superimposed way, where each sample is represented by two markers. The filled markers represent the coordinates in the features extracted from the copy number variables, and the open markers represent coordinates in the features extracted from the gene expression variables. Samples with different leukemia subtypes are shown with different colors. Without regularization (left panel), the extracted features, although being highly correlated, do not encode any biological information and are not generalizable to extract correlated information from the validation set (compare to the representation of the validation samples in the left panel of Figure 6). With maximal regularization (right panel), the agreement between the extracted features is weak, although they encode a large part of the variance in the data sets.*

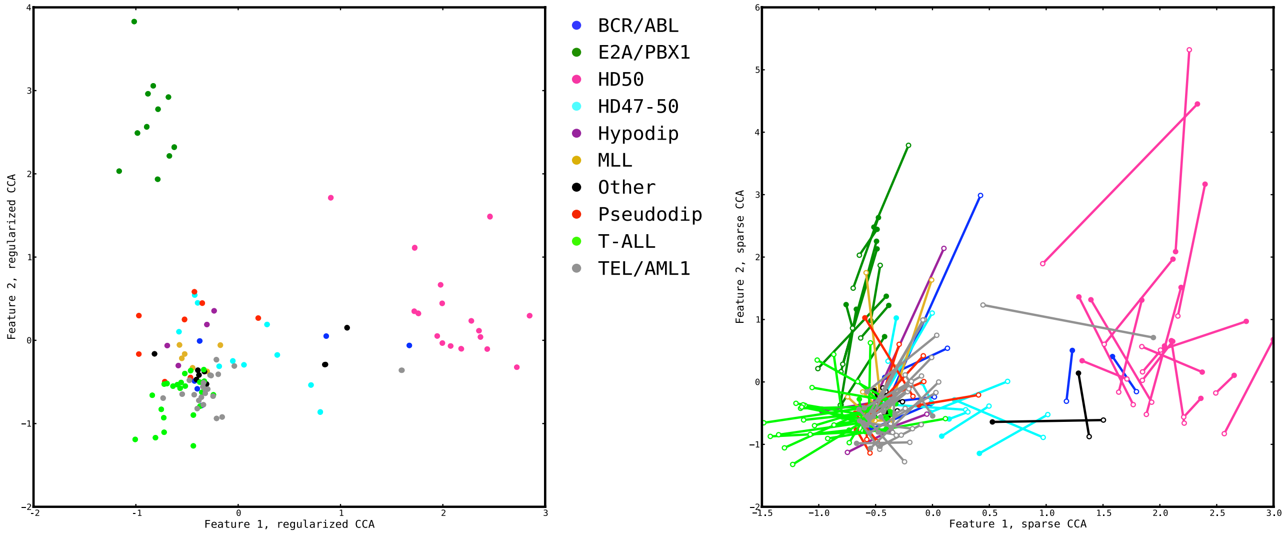

Supplementary Figure 3. *Representation of the samples from the tuning set in the features extracted from the tuning set using a subset of the variables, by regularized dual CCA (left panel,* $\tau_x = 0.75$*,* $\tau_y = 0.3$*) and the sparse CCA proposed by Witten* et al. *[1] (right panel). Apparently, the features extracted using regularized dual CCA have a higher correlation, as shown by the shorter distance between the points corresponding to each sample in the left panel. Different leukemia subtypes are shown with different colors.*

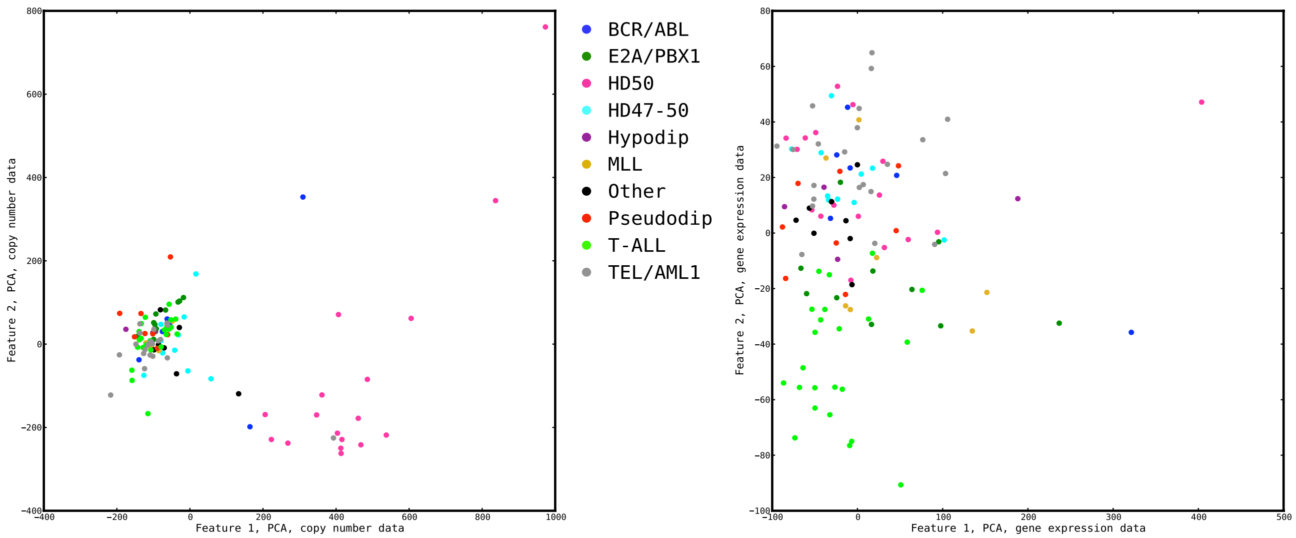

Supplementary Figure 4. *Representation of the samples from the tuning set by the projection onto the first two principal components (extracted from the tuning set) from the copy number data set (left panel) and gene expression data set (right panel), respectively. Samples with different leukemia subtypes are shown with different colors. The E2A/PBX1 subgroup, well separated from the rest using the integrative correlation-maximizing methods (Figure 5), is not emerging as a deviating group when the data sets are analyzed individually with PCA. The T-ALL subgroup (light green) has a specific gene expression pattern, and is discriminated from the rest using PCA on only the gene expression data (right panel). However, since these subjects do not have a specific copy number pattern, they are less discernible using CCA.*

References

[1] Witten DM, Tibshirani R, Hastie T: **A penalized matrix decomposition, with applications to sparse principal components and canonical correlation analysis.** *Biostatistics* 2009, **10**:515-534
